# The impact of parenting style, temperament and character on problematic internet and smartphone use among non-clinical female adolescents

**DOI:** 10.55730/1300-0144.5993

**Published:** 2024-06-11

**Authors:** Sadettin Burak AÇIKEL, Nur ÇETİN

**Affiliations:** 1 Department of Child and Adolescent Psychiatry, Ankara University School of Medicine, Ankara, Turkiye; 2 Autism Intervention and Research Center, Ankara University, Ankara, Turkiye; 3 Department of Child Development, Kalaba Vocational and Technical Anatolian High School, Turkish Ministry of National Education, Ankara, Turkiye

**Keywords:** Smartphone, behavior, internet addiction disorder, technology

## Abstract

**Background/aim:**

It is known that various temperament and character dimensions, and parenting styles could be associated with problematic internet and smartphone use. This study aimed to examine the association between temperament, character, and parenting styles and problematic smartphone and internet use in nonclinical female teenagers.

**Materials and methods:**

244 nonclinical female adolescent were included the study. Young’s Internet Addiction Test Short Form, Smartphone Addiction Scale, Junior Temperament and Character Inventory and Parenting Style Scale were used for collecting data.

**Results:**

Problematic smartphone use was significantly negatively correlated with cooperativeness character type, strictness/supervision, and acceptance/involvement subscales of the parenting style scale. A significant correlation was found between problematic internet use and acceptance/involvement, strictness/supervision subscales of Parenting Style Scale. In ANOVA analysis, it was found that the authoritative parenting style was associated with less problematic use, and the neglectful, authoritarian, and indulgent parenting styles were associated with increased problematic use. A negative correlation was found between the cooperativeness character style and problematic smartphone use.

**Conclusion:**

According to our results, parenting style is more important for problematic internet and smartphone use among female adolescents than temperament and character.

## 1. Introduction

In recent years, the active use of smartphones and the internet in many areas such as participating on social networks, online education, shopping, and travel, as well as communication, has increased their influence in our lives. Peak smartphone and internet use is observed in 14–20 ages [1]. Therefore, adolescents are vulnerable to the negative effects because of both excessive usage and characteristics of adolescence such as difficulties about impulse control and cognitive flexibility. Problematic internet and smartphone use can lead to various problems in the lives of adolescents due to both structural features and intensive use. [2]. These could be listed as negative impacts on daily life function, social and family relationships, and emotional instability, sleep, and appetite [3]. There are relevant studies on this issue. Some of these studies have investigated the effects of problematic smartphone use on appetite and sleep. In one of these studies, problematic smartphone use was found to have negative effects on sleep quality. In addition, it was emphasized that bedtime procrastination may be a mediator between problematic smartphone use and sleep quality [4]. Another study revealed a strong correlation between eating disorders, eating behaviors, and lifestyle choices, and problematic smartphone use [5]. In one study, problematic internet use was investigated in relation to coping mechanisms and functioning. The findings demonstrated the relationship between problematic internet use and negative coping strategies, avoidant behavior, preferring a virtual life, escapism, and the length of time spent on social media, as well as impairment in social media-related functionality [6]. When all these data are evaluated, adolescence is a period in which problematic smartphone and internet use is both common and problems related to them are frequently experienced.

Although DSM-5 presents several regulations were made about internet gaming disorder to conditions for further study [7], the diagnostic criteria of problematic internet and smartphone use are not clearly defined. Problematic internet use generally refers to the excessive and problematic use of the internet. [8]. Problematic smartphone use also is basically defined as excessive use of smartphones by Lin et al. [9]. The prevalence of these two conditions has been increased in whole world among adult and adolescent groups [10]. This has become an important global mental health problem and attracts attention. These behavioral addictions comprise four essential elements: tolerance, withdrawal, obsessive symptoms, and functional impairment like other addictions [9]. Most smartphone users also use the internet extensively, smartphone ownership is strongly related to internet connectivity, and these two situations are often comorbid. [11]. Early detection is important for problematic internet and smartphone use and this approach should take individual and environmental risk factors into consideration. There are several risk factors that lead up to problematic internet and smartphone use. These factors could be classified as individual risk factors and environmental risk factors. It has been found that several psychiatric disorders [12–14], and temperament dimensions [15] could be related with problematic internet use.

According to Cloninger’s theory of temperament and personality, temperament contains four dimensions; novelty seeking (NS), harm avoidance (HA), reward dependence (RD), and persistence (P). Novelty seeking is characterized by traits such as being easily excited, impulsive, inclined to explore, and having a quick temper. Harm avoidance refers to an individual’s tendency to be cautious, apprehensive, and excessively pessimistic. Reward dependence involves maintaining behaviors that have been previously reinforced and being sensitive, sentimental, and reliant on the approval of others. Persistence refers to the ability to persevere in behavior despite a lack of reward and feelings of fatigue [16,17]. Several studies have explored the association between temperament and internet addiction/problematic internet use in adolescent and adult populations. Ko et al. conducted a study on 216 college students (132 males and 84 females) with a mean age of 21.45 ± 2.05 (range: 18–27) and discovered, for instance, that problematic internet use was associated with high levels of novelty seeking and harm avoidance, but low levels of reward dependence [18]. Dalbudak et al. discovered that university students with problematic internet use exhibited higher novelty seeking scores and lower self-directedness and cooperativeness scores [15]. In a recent study, low levels of novelty seeking, and high levels of reward dependence and harm avoidance was associated with problematic smartphone use in the adult population [19]. In another study conducted on 1109 adults investigating problematic internet use and personality traits. The study found a significant correlation between extraversion, openness to experience, and problematic internet use. The adjustment models revealed a positive correlation between these characteristics and being unmarried as well as having a higher level of education [20]. In another recent study, problematic internet use was examined in psychiatric inpatients and interpreted from a sex perspective. This study was conducted on a group of 104 adolescents, with 69 of them being girls. According to results, boys exhibited a higher frequency of engaging in gaming activities, while girls showed a greater inclination towards social networking. Sex-specific analyses revealed disparities between males and females. Girls diagnosed with PIU exhibited significantly elevated levels of internalizing and externalizing problems and behaviors, as well as novelty seeking and transcendence. Conversely, they demonstrated lower levels of persistence, self-directedness, and cooperativeness compared to girls without PIU. Boys who have PIU exhibited notably elevated scores in internalizing problems and self-transcendence, while showing reduced scores in harm avoidance compared to boys who do not have PIU. It was mentioned that sex could be a significant factor in PIU. Sex-specific disparities in application usage, as well as symptomatic, temperament, and character traits, necessitate a sex-specific approach in the integration of prevention and treatment [21]. To our best knowledge, there is no study that explore the association between problematic internet and smartphone use and temperament in adolescents simultaneously in nonclinical sample.

Parenting style is considered an important environmental factor that influence problematic internet and smartphone use. In a recent study about this subject, parental responsiveness was negatively, maternal strictness was positively associate with problematic internet use. A combination of maternal authoritarianism and paternal neglectfulness was linked to an increase in problematic internet use, whereas authoritative parenting styles exhibited by both parents reduced problematic internet use [22]. Therefore, parenting style could be important in problematic internet and smartphone use. To our best knowledge, there is no study that explore the association between problematic smartphone use and parenting style in adolescents.

Problematic internet and smartphone use are behavioral problems caused by neurobiological and environmental factors. Recent studies have shown that executive functions and reward cycles are affected in these cases [23]. In the current study, it was aimed to investigate the effect of temperament and character traits, which are innate characteristics of the individual, and parenting skills, which are environmental factors, on internet and smartphone use. The purpose of this study was to investigate the effect that parenting style and temperament have on problematic internet and smartphone use in female adolescents. To homogenize the sample, these variables were explored in one sex. It was hypothesized that both temperament and parenting style could influence adolescents’ problematic internet and smartphone use. It was also hypothesized that there could be an association between different parenting styles, temperament, and problematic internet and smartphone use.

## 2. Materials and methods

In this study, 244 female students have been included. The study was conducted in a public school approved by the Ministry of National Education and attended by youngsters from all socioeconomic backgrounds. The reason for selecting a public school was to ensure that children from households with similar socioeconomic levels participated in the study. All the participants were selected from the same high school in Ankara, which is the capital of Türkiye. The criterion for eligibility to participate in the study was that they volunteered. Students were initially informed about the study. Subsequently, a researcher presented the study’s specifics to the high school students and solicited participation. With the exception of those who were not enrolled in classes or were unable to attend due to personal circumstances, nearly all the invited students were included in the study. Having any chronic medical conditions, undergoing medical treatment, having any present psychological disorder have been defined exclusion criteria. The questionnaires were completed in small groups during class (almost 20 students) and took approximately 30 min. Turkish versions of all the questionnaires were used. The protocol for this study (2012-KAEK-15/2087) was approved by both the Turkish Ministry of National Education and the Keçiören Training and Research Hospital Ethics Committee, and each participant provided informed consent.

### 2.1. Tools

Sociodemographic Data Form: This form which includes questions about age, family characteristics, and class was prepared by the authors. Questions about smartphone use were used (Do you personally have a smartphone? If yes, since when? How many hours a day do you spend with your smartphone? What is the main purpose of your smartphone use? Do you play online games? If yes, how many hours do you play in a day? Which social networks do you participate on?) to determine the characteristic the partcipants’internet and smartphone use.

Young’s Internet Addiction Test Short Form (YIAT-SF): This scale was developed by Young and shortened by Pawlikowski et al.[24]. The scale consists of 12 items 5-point Likert (1 = Never, 5 = Very often) type scale. There are no reverse items in the scale. The total score is obtained by summing the scores obtained from all items. High scores indicate increased levels of internet addiction. The validity and reliability adaptation study of the Turkish version was performed on adolescents. The Cronbach’s alpha coefficient obtained in the reliability study of the Young Internet Addiction Test Short Form was 0.91 for university students and 0.86 for adolescents. When the test-retest reliability of the scale was examined, the correlation coefficient between the two applications was 0.93 for university students and 0.86 for adolescents. [25].

Smartphone Addiction Scale-Short Version (SAS-SV): The SAS-SV is a self-assessment tool that measures adolescents’ “smartphone addiction” using a single subdimension. The original form of the scale had ten items with a 6-point Likert scale (1: strongly disagree, 6: strongly agree). This scale produces scores ranging from 10 to 60. The cut-off score was defined 33 for females and 31 for males in the original study. The validity and reliability adaptation study of the Turkish version was performed on adolescents. The item-total correlation coefficients of the items in the scale ranged between 0.40–0.75. The Cronbach’s alpha coefficient of the 10-item SAS-SV was 0.867 [26].

Junior Temperament and Character Inventory-Revised (J-TCI-R). Luby et al. developed the scale based on Cloninger’s biopsychosocial model to assess temperament and character characteristics in children and adolescents [27]. It comprises 125 questions divided into six 5-point Likert scales. The inventory’s four subscales represent temperament traits, including novelty seeking, harm avoidance, reward dependency, and persistence. The remaining two subscales are character dimensions: self-directedness and cooperativeness. According to the Turkish adaptation study, the scale is valid and trustworthy for measuring the temperament and character traits of children and adolescents. Kose et al. modified the scale into Turkish. The Cronbach’s alphas for the temperament and character subscales of J-TCI-R ranged from 0.60 to 0.75. The correlations between the baseline and one-month post administration of J-TCI-R were both highly and statistically significant. After conducting factor analysis on all the subscales, four out of six factors were retained. This study represents the first known analysis of the psychometric properties and factorial construct of the J-TCI-R [28].

Parenting Style Scale (PSS): The PSS is a 24-item Likert type scale that contains three subscales: acceptance/involvement, strictness/supervision, and psychological autonomy [29]. The adolescent completes the scale to determine parenting styles. The validity and reliability adaptation study of the Turkish version was performed on adolescents. The reliability coefficients and internal consistency coefficients for high school students were 0.82 and 0.70 for the acceptance/involvement subscale, 0.88 and 0.69 for the strictness/supervision subscale, and 0.76 and 0.66 for the psychological autonomy subscale, respectively [30]. The acceptance/involvement subscale aims to measure how much children perceive their parents as caring, involved, and participatory. The strictness/supervision subscale aims to measure the degree to which children perceive their parents as controlling with strict supervision. The psychological autonomy subscale aims to measure the extent to which parents practice democratic attitudes and encourage the expression of the individuality of the child. The scale has four parenting categories: authoritative, neglectful, authoritarian, and indulgent, which are scored according to scores obtained from the subscales.

### 2.2. Statistical analysis

IBM SPSS Statistics for Windows 24.0 (IBM Corp., Armonk NY, USA) was used for the analyses, and descriptive analyses were performed to determine the frequencies of the tested variables. Jamovi software was used to create the figures [31]. Frequency and percentage were used in the analysis of categorical data as descriptive statistics and mean, and standard deviation were used in the analysis of continuous data. Normality of data was evaluated according to skewness and kurtosis values. When the assumption of normal distribution was realized, the Independent Sample’s t test was used to evaluate the differences in the scores between groups, and Pearson correlation analyses were used to examine the relationships between the variables. One-way analysis of variance (ANOVA) and Tukey tests were used in the comparison of more than two groups of continuous data in the study. In the comparison of more than two groups of continuous data, One-way ANOVA and post hoc tests were used as Tukey because the variances were homogeneous.

## 3. Results

The study sample was consisted of 244 female adolescents. The mean age of sample was 16.4 ± 0.93 years. Of the participants, 93.7% (n = 227) had a smartphone. The mean duration of time they had one was 3.76 ± 1.62 years and the mean duration of daily use was 2.77 ± 0.93 h/day. Moreover, 84% (n = 205) of the participants had mobile data plan and the mean mobile data limit on their plans was 4.89 ± 3.34 GB/month. The participants were asked, “what is the main purpose of using smartphone?” to which 59.4% (n = 145) stated that they used it most frequently for surfing social media. This rate was 23.8% (n = 58) for surfing the web, 10.7% (n = 26) for only communication, and 6.1% (n = 15) for playing games. Moreover, 21.7% (n = 53) of the participants stated that it was for playing online games and the mean time they spent doing this was 1.80 ± 1.21 h/day. Instagram was the most preferred social media network followed by YouTube, TikTok, Twitter, and Facebook. These descriptive statistics are presented in Table 1.

The mean SAS-SV scores of sample was 30.4 ± 11.13 and YIAT scores was 26.9 ± 8.89. The mean SAS-SV and YIAT scores were significantly different between participants using Instagram and TikTok and those who did not. This difference was not observed for theother social networks used. The mean SAS-SV score was 31.52 ± 11.20 among Instagram users whereas 25.35 ± 9.31 among participants which do not use Instagram. The mean YIAT scores score was 27.65 ± 8.81 among Instagram users whereas 23.41 ± 8.55 among participants which do not use Instagram. There was a significant difference in terms of the SAS-SV score (t = 0.314, p = 0.001) and YIAT score (t = 2.850, p = 0.005). The mean SAS-SV score was 34.29 ± 10.27 among TikTok users whereas 29.01 ± 11.13 among participants which do not use TikTok. There was a significant difference in terms of the SAS-SV score (t = 3.373, p = 0.001).

**Figure 1:**
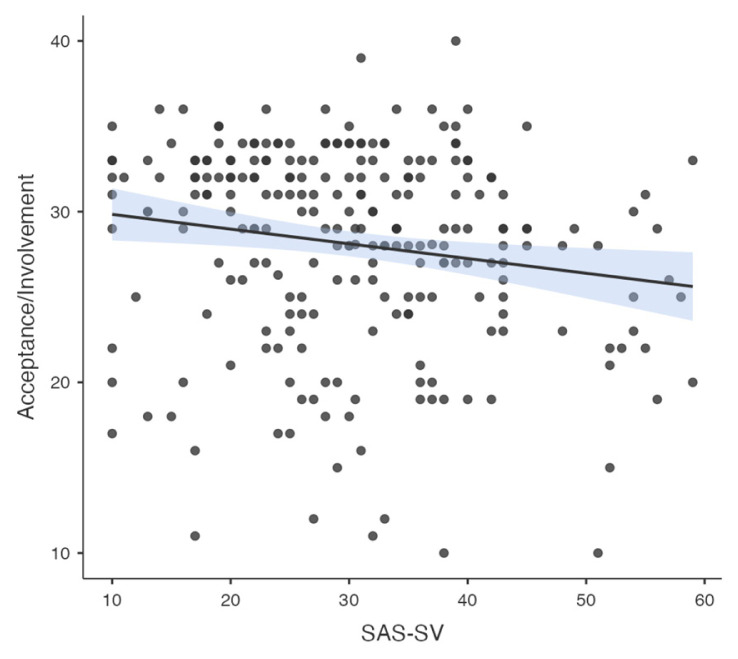
Correlation of SAS-SV and acceptance/involvement.

**Figure 2:**
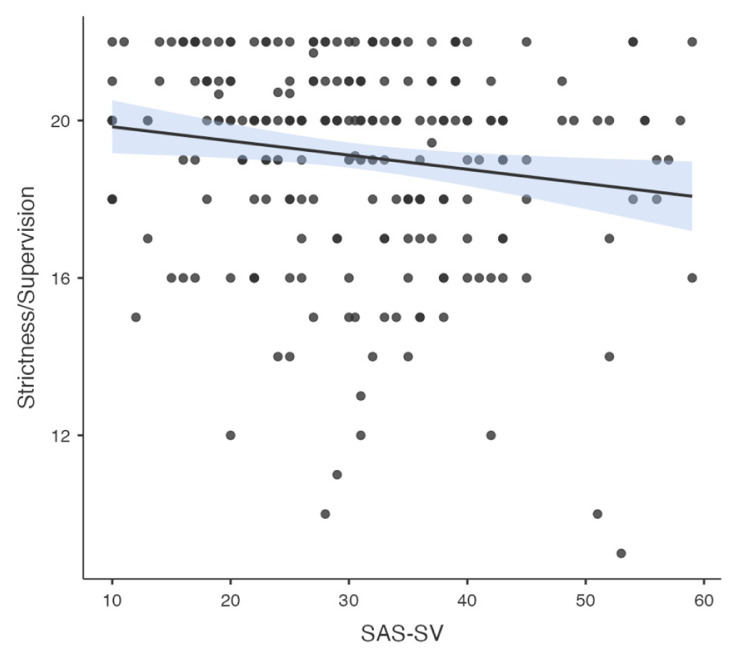
Correlation of SAS-SV and strictness/ supervision.

**Figure 3:**
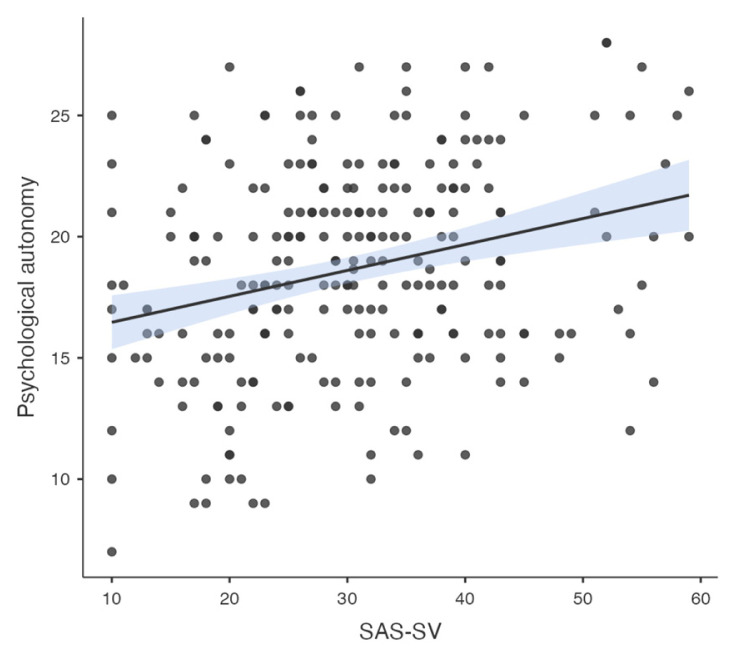
Correlation of SAS-SV and psychological autonomy.

**Figure 4:**
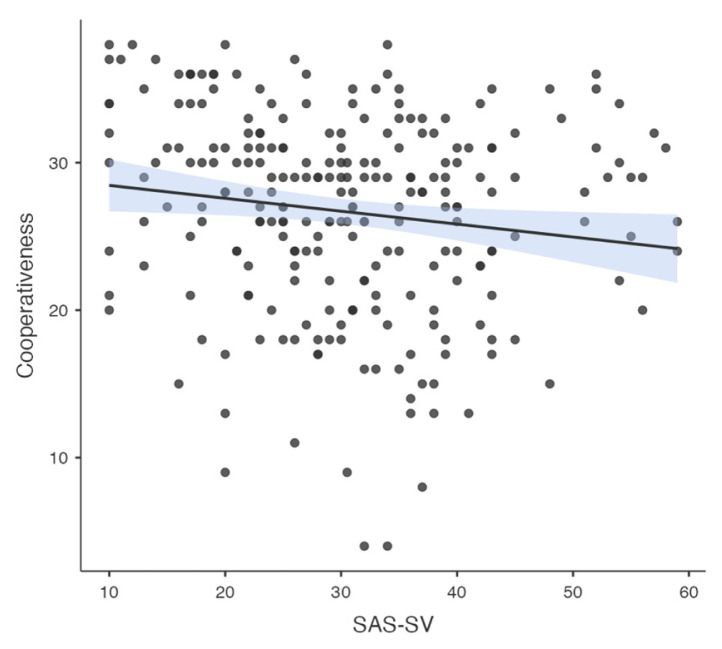
Correlation of SAS-SV and cooperativeness.

**Figure 5:**
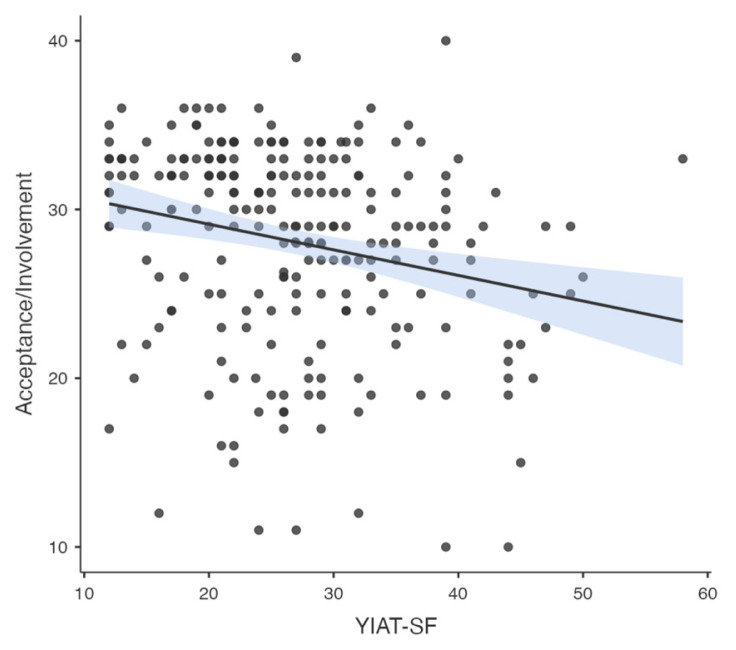
Correlation of YIAT-SF and acceptance/involvement.

**Figure 6:**
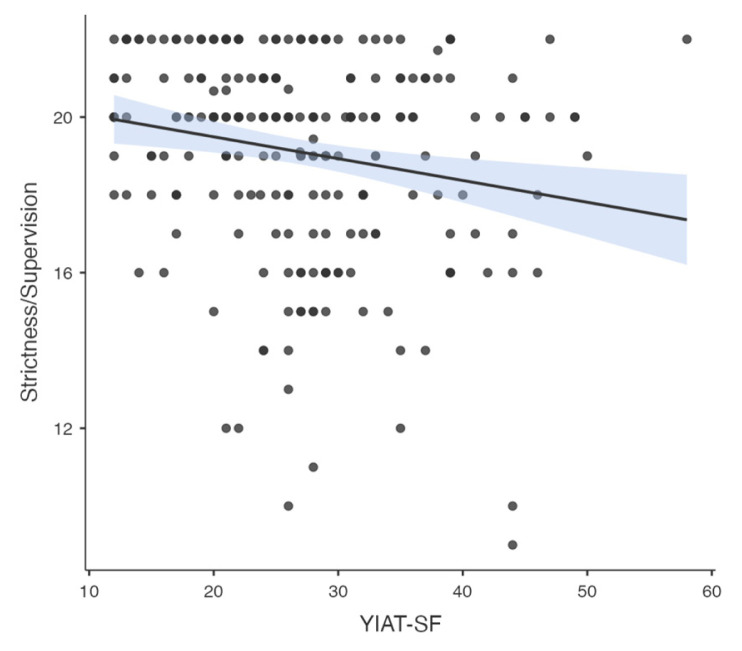
Correlation of YIAT-SF and strictness/supervision.

**Figure 7:**
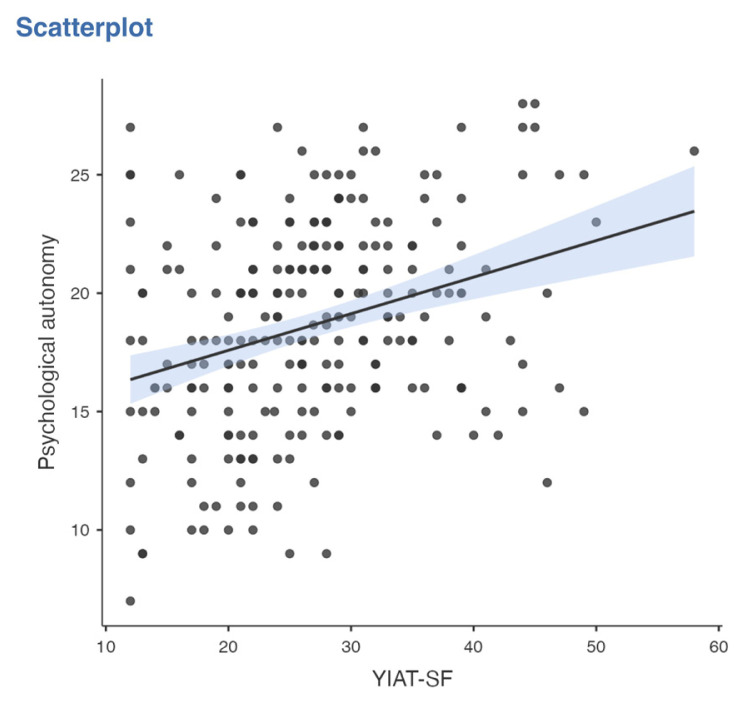
Correlation of YIAT-SF and psychological autonomy.

Pearson correlation analysis was used to determine the association between all temperament and character dimensions, parenting style, problematic smartphone and internet use. A significant correlation was found between problematic smartphone use and internet use (R = 0.776, p < 0.001). A significant correlation was found between problematic smartphone use and acceptance/involvement (R = −0.164, p = 0.01), strictness/supervision (R = −0.156, p = 0.015 and psychological autonomy (R = 0.273, p < 0.001) subscales of Parenting Style Scale and cooperativeness character style (R = −0.145, p = 0.024). Significant correlation was found between problematic internet use and acceptance/involvement (R = −0.231, p < 0.001), strictness/supervision (R = −0.194, p = 0.002 and psychological autonomy (R = 0.315, p < 0.001) subscales of PSS. There was no significant correlation between problematic internet use and temperament and character subdimensions. The results of correlation analysis are given in Table 2 and Figures 1–7.

ANOVA was used to determine whether problematic smartphone use, and problematic internet use differed based on the parenting styles. Problematic smartphone use and problematic internet use levels were compared among authoritative, neglectful, authoritarian and indulgent parenting styles. The mean smartphone addiction score was 26.79 ± 10.28 in the authoritative parenting group, 32.05 ± 11.44 in neglectful parenting group, 36.67 ± 10.91 in authoritarian parenting group and 32.38 ± 9.62 in indulgent parenting group. The difference between the groups was significant (F = 8.671, p < 0.001). Post hoc analyses using the Tukey post hoc criterion for significance indicated that in the authoritative parenting group, the SAS-SV scores were significantly lower than in all the other parenting style groups. The difference between the other three parenting style groups was not significant.

**Figure 8:**
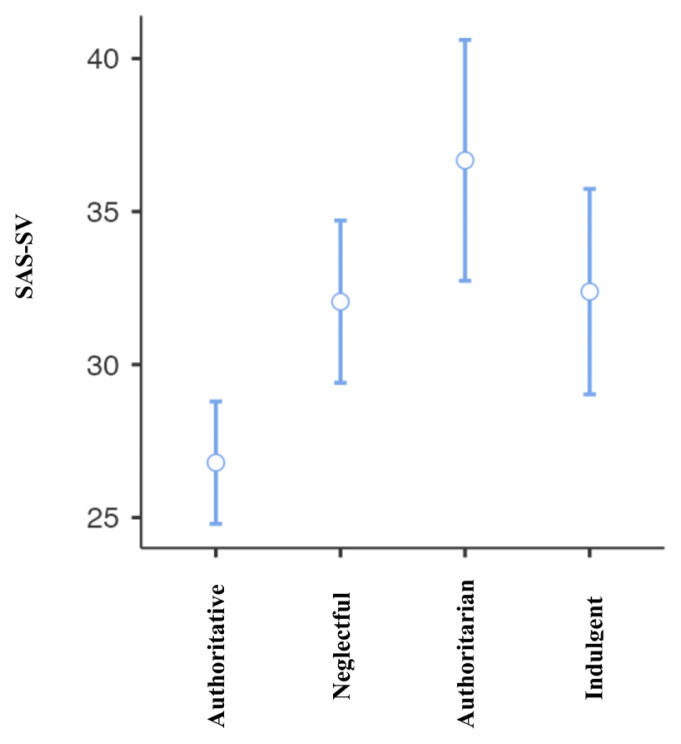
Anova results of SAS-SV.

The mean YIAT score was 24.09 ± 8.79 in the authoritative parenting group, 28.09 ± 8.27 in the neglectful parenting group, 31.28 ± 9.25 in the authoritarian parenting group, and 27.20 ± 7.57 in the indulgent parenting group. The difference between the groups was significant (F = 7.921, p < 0.001). Post hoc analyses using the Tukey post hoc criterion for significance indicated that in the authoritative parenting group YIAT scale levels were significantly lower than in the neglectful and authoritarian parenting style groups. The ANOVA results are given in Table 3 and 4 and Figures 7–8.

## 4. Discussion

The objective of this study was to examine the association between problematic smartphone and internet use and temperament, character, and parenting style. We have found a significant negative correlation between parental acceptance and supervision and smartphone and internet addiction. Also, we have found a significant positive correlation between psychological autonomy and problematic smartphone and internet use. We could not find any significant correlation between temperament and character dimensions and problematic smartphone and internet use except the association between smartphone addiction scores and cooperativeness character style. Also, we have found increased problematic smartphone and internet use scores among Instagram and TikTok users. In addition, we have found significantly lower problematic smartphone and internet use scores authoritative parenting group in One-way ANOVA. The current study is the first to investigate the effect of parenting style, temperament, and character dimensions on both problematic smartphone and internet use in a nonclinical sample.

Among adolescents, using of smartphones is increasing gradually and becoming a serious problem [32]. This excessive use of smartphones could cause conflicts with parents, and with teachers with inattention to schoolwork, the interruption of social interaction, or sleep disturbances, which may be attributable to smartphone overuse [33]. Problematic smartphone and internet use symptoms could force adolescents to continue to be online and result in unpleasant emotions and psychological difficulties [34]. The current study revealed that increased problematic smartphone and internet use among Instagram and TikTok users. We believe that these social networks may force adolescents to be online more and lead to more problematic smartphone and internet use due to their features such as frequent sharing, story sharing and fast-changing contents.

Numerous studies have investigated the connection between problematic internet use and parenting. A recent study discovered that the permissive parenting style had a moderating influence on the relationship between worry and problematic internet use in youngsters. The study proposed that there is a bigger positive correlation between concern and problematic internet use when the level of permissive parenting is higher [35]. In a separate investigation, Li et al. discovered a positive correlation between problematic internet use and parental discipline, rejection, excessive intervention, and academic pressure. Conversely, they identified a negative association between problematic internet use and favorable teacher-student interactions, positive schoolmate relationships, and cognitive function scores [36]. In a similar study, Lukavska et al. found a negative association between parental responsiveness and problematic internet use, and positive association between maternal strictness and problematic internet use. They found that the authoritative parenting style in both parents is associated with decreased problematic internet use [37]. Similarly, we have found decreased problematic smartphone and internet use among authoritative parenting group. In another study conducted in our country, Karaer et al. found inadequate in acceptance/involvement, supervision/monitoring and lower parental strictness/supervision is associated with problematic internet use [38]. We have also found similar results with this study. All these findings could show that optimal parenting style can be protective in terms of problematic smartphone and internet use. To the best of our knowledge, this study is the first to investigate problematic smartphone and internet use at the same time.

Numerous studies have been conducted to examine the correlation between temperament and problematic internet use. Most of the research was carried out on adult. A recent meta-analysis suggested that traits associated with avoidance of injury and dependence, novelty seeking, and obsessive-absorption are significant risk factors for problematic internet use [39]. Hanafi et al. additionally demonstrated that among medical students, problematic smartphone use is associated with elevated levels of reward dependence and harm avoidance, as opposed to low levels of novelty seeking [40]. A separate study involving adolescents diagnosed with attention-deficit/hyperactivity disorder (ADHD) found that high levels of novelty seeking and low levels of overprotection from parents were strongly linked to problematic smartphone use scores in the ADHD group. On the other hand, high levels of novelty seeking, and low levels of persistence were strongly linked to problematic smartphone use scores in the group without ADHD [41]. In our study we have found a significant negative correlation between problematic smartphone use scores and cooperativeness character style. Dalbudak et al. have found higher novelty seeking (NS) scores and lower self-directedness (SD) and cooperativeness (C) scores in the moderate/high problematic internet use group [42]. In addition to this finding, Pettorruso et al. have found low self-directedness and cooperativeness among patient with gambling disorder [43]. In light of these findings, low cooperativeness could be related with addictive behaviors and this finding was consistent with the literature.

There are studies showing that internet applications are used for different purposes between sexes. Research has shown that women are more actively involved in activities such as information search, watching videos, and communication through platforms and social networks. In addition, female adolescents with problematic internet use exhibit a complex combination of personality traits, characterized by both high levels of social and emotional impairment, as well as extraversion and positive affect. [44]. In a recent study, it was found that female adolescents diagnosed with problematic internet use exhibited significantly elevated levels of internalizing and externalizing problematic behaviors, novelty seeking, and transcendence. Conversely, they displayed lower levels of persistence, self-directedness, and cooperativeness compared to those without problematic internet use [45]. Like these results, a significant negative correlation was found herein between problematic smartphone use and cooperativeness among female adolescents. The similar results in the current study suggest that the factors associated with problematic internet and smartphone use may differ between sexes.

There are several limitations of the current study that should be considered when interpreting the results obtained. First, the study sample consisted of only female adolescents. This limitation could have influenced the result, especially in terms of the temperament and character dimensions. Second, diagnostic interviews were not conducted because of the design of the study. Due to this limitation, use of the term ‘smartphone and internet addiction’ was avoided, instead of this, ‘problematic internet and smartphone use’ was used. Moreover, any comorbid psychiatric diagnosis among the participants could have interfered with the results. Third, the parenting style was obtained from an adolescent rated scale. This could have interfered with the results because of the potential bias. We did not control psychiatric symptom dimensions such as depression, anxiety, and attention deficit hyperactivity disorder. This could influence our result. And last, but not least, parameters such as the use of tablets and computers were not considered separately. This could be listed as a limitation.

This study also has some practical implications. A significant negative correlation was found between parental acceptance and supervision and smartphone and internet addiction, resulting in significantly lower smartphone and internet addiction scores in the authoritative parenting group via One-way ANOVA. This finding may suggest that internet addiction may decrease as parental acceptance and supervision skills increase in the relationship between adolescents and parents. Additionally, authoritative parenting could be protective against smartphone and internet addiction. Moreover, a significant positive correlation was found between psychological autonomy and smartphone and internet addiction. The psychological autonomy dimension we used aims to measure the extent to which parents apply a democratic attitude and the extent to which children are encouraged to express their individuality. This result can be interpreted that if adolescents gain more autonomy, their control over internet and smartphone use may decrease and parental supervision may be more important in behavioral addictions. A significant negative correlation was found between problematic smartphone use and cooperativeness character style. This finding can be interpreted as the decrease in smartphone addiction as the adolescents’ cooperation skills increase. Also found was greater smartphone addiction in TikTok and Instagram users. This finding may be explained by the fact that these platforms are used more intensively among adolescents and have fast changing and attention-grabbing content. Undoubtedly, the fact that the study was cross-sectional and did not include a follow-up cause along the fact that these findings cannot provide a clear causality.

As a result, parenting style and some character style could be an important component of adolescents’ problematic smartphone and internet use. This study is important because it is the first study to investigate the association between parenting style, temperament and character style, problematic smartphone, and internet use at the same time. It is recommended that future studies be designed with larger samples, including both sexes, and include psychiatric evaluations of the parents in the analysis.

## Figures and Tables

**Figure 9:**
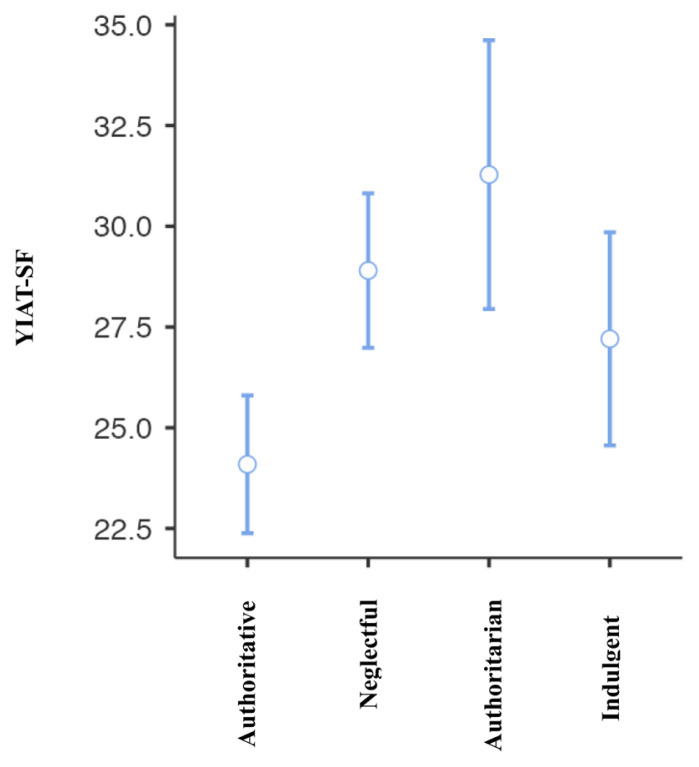
Anova results of YIAT-SF.

**Table 1:** Descriptive statistics.

	Mean		SD	
**Age (year)**	16.4		0.93	
**Duration of having smartphone (year)**	3.76		1.62	
**Duration of daily usage (hour)**	2.77		0.93	
**Limit of mobile data plan (gigabyte)**	4.89		3.34	
	**Yes**		**No**	
	**N**	**%**	**N**	**%**
**Having a smartphone**	227	93	17	7
**Having mobile data plan**	206	87.7	29	12.3
**Playing online games**	53	78	188	22
**Instagram**	202	82.2	42	17.2
**YouTube**	143	58.6	101	41.4
**TikTok**	67	27.5	177	72.5
**Twitter**	65	26.6	179	73.4
**Facebook**	59	24.2	185	75.8

**Table 2:** Correlations between problematic smartphone and internet use and the parenting styles.

	SAS-SV	YIAT-SF	Acceptance/involvement	Strictness/Supervision	Psychological autonomy	Novelty seeking	Harm avoidance	Reward dependence	Persistence	Self-directedness
SAS-SV										
YIAT-SF	0.776**									
Acceptance/involvement	−0.164*	−0.231**								
Strictness/supervision	−0.156*	−0.194**	0.526**							
Psychological autonomy	0.273*	0.315**	−0.163*	−0.024						
Novelty seeking	−0.044	0.002	0.084	0.068	0.006					
Harm avoidance	−0.055	−0.012	0.052	0.049	0.010	0.800**				
Reward dependence	−0.032	0.049	0.086	0.041	0.039	0.759**	0.710**			
Persistence	−0.122	−0.079	0.135*	0.070	−0.028	0.783**	0.749**	0.628**		
Self-directedness	−0.104	−0.077	0.164*	0.076	−0.044	0.787**	0.759**	0.728**	0.777**	
Cooperativeness	−0.145*	−0.075	0.109	0.079	−0.022	0.784**	0.774**	0.758**	0.737**	0.765**

** Correlation is significant at 0.01 (2-tailed).

* Correlation is significant at 0.05 (2-tailed).

**Table 3:** Data comparison of the smartphone and internet addiction by parenting style.

		N	Mean	SD	F	p*
**SAS-SV**	Authoritative parenting	104	27.79	10.28	**8.671**	**<0.001**
	Neglectful parenting	74	32.05	11.44		
	Authoritarian parenting	32	36.67	10.91		
	Indulgent parenting	34	32.38	9.62		
	Total	244	30.46	11.14		
**YIAT-SF**	Authoritative parenting	104	24.09	8.79	**7.921**	**<0.001**
	Neglectful parenting	74	28.90	8.27		
	Authoritarian parenting	32	31.28	9.25		
	Indulgent parenting	34	27.21	7.58		
	Total	244	26.93	8.90		

* One-Way ANOVA

**Table 4:** Tukey HSD post-hoc comparisons.

						95% Confidence interval	
	Parenting style	Parenting style	Mean diff (I–J)	Std. error	Sig.	Lower bound	Upper bound
**SAS-SV**	Authoritative parenting	Neglectful parenting	−5.26111*	1.61870	0.007	−9.4489	−1.0733
		Authoritarian parenting	−9.87901	2.15162	<0.001	−15.4455	−4.3125
		Indulgent parenting	−5.58890	2.10267	0.041	−11.0288	−0.1490
**YIAT-SF**	Authoritative parenting	Neglectful parenting	−4.80947	1.29903	0.002	−8.1702	−1.4487
		Authoritarian parenting	−7.18967	1.72671	<0.001	−11.6569	−2.7225
		Indulgent parenting	−3.11430	1.68743	0.255	−7.4799	1.2513
